# Cardioinhibitory syncope with asystole during nitroglycerin potentiated head up tilt test: prevalence and clinical predictors

**DOI:** 10.1007/s10286-022-00864-3

**Published:** 2022-05-06

**Authors:** Vincenzo Russo, Erika Parente, Anna Rago, Angelo Comune, Nunzia Laezza, Andrea Antonio Papa, Celeste Chamberland, Thao Huynh, Paolo Golino, Michele Brignole, Gerardo Nigro

**Affiliations:** 1 Cardiology and Syncope Unit, Department of Translational Medical Sciences, University of Campania “Luigi Vanvitelli”-Monaldi Hospital, Naples, Italy; 2 Department of History and Philosophy, Roosevelt University, Chicago, USA; 3 Division of Cardiology, McGill Health University, McGill University, Montreal, Canada; 4 Faint & Fall programme, Department of Cardiology, IRCCS Istituto Auxologico Italiano - San Luca Hospital, Milan, Italy

**Keywords:** Syncope, Asystole, Predictors, Age, Gender, Reflex neurally mediated

## Abstract

**Aims:**

The aim of our study was to evaluate the prevalence and clinical predictors of cardioinhibitory (CI) responses with asystole at the nitroglycerin (NTG)-potentiated head-up tilt test (HUTT) in patients with a history of syncope admitted to a tertiary referral syncope unit.

**Methods:**

We retrospectively evaluated all consecutive patients who underwent NTG-potentiated HUTT for suspected reflex syncope at our institution from March 1 2017 to May 1 2020. The prevalence of HUTT-induced CI syncope was assessed. Univariate and multivariate analyses were performed to test the association of asystolic response to HUTT with a set of clinical covariates.

**Results:**

We enrolled 1285 patients (45 ± 19.1 years; 49.6% male); 368 (28.6%) showed HUTT-induced CI response with asystole. A multivariate analysis revealed that the following factors were independently associated with HUTT-induced CI syncope: male sex (OR 1.48; ConInt 1.14–1.92; *P* = 0.003), smoking (OR 2.22; ConInt 1.56–3.115; *P* < 0.001), traumatic syncope (OR: 2.81; ConInt 1.79–4.42; *P* < 0.001), situational syncope (OR 0.45; ConInt 0.27–0.73; *P* = 0.002), and the use of diuretics (OR 9.94; ConInt 3.83–25.76; *P* < 0.001).

**Conclusions:**

The cardioinhibitory syncope with asystole induced by NTG-potentiated HUTT is more frequent than previously reported. The male gender, smoking habit, history of traumatic syncope, and use of diuretics were independent predictors of HUTT-induced CI responses. Conversely, the history of situational syncope seems to reduce this probability.

## WHAT’S NEW?

- Cardioinhibitory response with asystole to NTG-potentiated HUTT is more frequent than previously reported
- Male gender, smoking habit, history of traumatic syncope, and use of diuretics were independently predictors of HUTT-induced cardioinhibitory syncope
- Situational syncope reduced the probability of cardioinhibitory response to HUTT.

## Introduction

The head-up tilt test (HUTT) is a useful and necessary diagnostic tool for patients with suspected reflex syncope after initial clinical assessment [1]. The overall positivity rate is 66%; however, it changes according to the different HUTT protocols and clinical features of anamnestic syncope [2]. The prevalence of HUTT-induced cardioinhibitory syncope ranges from 4.3% to 19% in patients with unexplained syncope undergoing HUTT [3–6]**;** however, these percentages refer to heterogeneous cohorts for age, inclusion criteria, and HUTT protocols. The evidence of an asystolic response to HUTT is predictive of spontaneous asystolic syncope [7], and is considered a marker for a priori identifying subjects who may benefit from pacemaker therapy [8]. Therefore, the identification of patients at increased risk of asystolic syncope might be useful for fast-track access to HUTT, in order to confirm the diagnosis and for early detection of those in need of pacemaker therapy. The aim of our study was to evaluate the prevalence and clinical predictors of cardioinhibitory response with asystole in nitroglycerin (NTG)-induced HUTT in patients with a history of syncope admitted to a tertiary referral syncope unit.

## Materials and methods

We retrospectively evaluated 1335 consecutive patients who underwent HUTT for suspected reflex syncope at the Syncope Unit of the University of Campania “Luigi Vanvitelli”—Monaldi Hospital of Naples, Italy from March 1 2017 to May 1 2020. The HUTT was performed according to the “Italian Protocol” with a supine pre-tilt phase of 10 min, no venous cannulation, a passive phase of tilt of 20 min at 70°, and, in cases of negativity, a NTG challenge with a fixed dose of 300 µg sublingually administered with the patient in the upright position. During the whole duration of the HUTT, continuous electrocardiographic monitoring and non-invasive beat-to-beat arterial blood pressure measurement (Task Force^®^ monitor; CNSystem, Graz, Austria) was performed. The HUTT continued until complete lack of consciousness (LOC) occurred, indicated by a lack of response to vocal stimuli, loss of muscle tonus, and jerking movements, whichever occurred first, or completion of the protocol, 20 min after the NTG administration; the time occurring for tilting down the motorized tilt table was 12 s. The responses were classified according to the new VASIS classification, also suggested by the 2018 ESC Guidelines [9].

In particular, a cardioinhibitory (CI) response was defined as syncope occurring in the presence of a ventricular pause of > 3 s (VASIS type IIB response); a mixed response was defined as syncope occurring in the presence of bradycardia and hypotension (including both VASIS type I and IIA responses); a vasodepressor (VD) response was defined as syncope occurring during hypotension with no or slight heart rate decrease (< 10 bpm) (VASIS type III response). We excluded all patients with HUTT-induced orthostatic hypotension or intolerance without syncope (*n* = 18), incomplete baseline information (*n* = 24), or without consent for data collection (*n* = 8). Thus, a total of 1285 patients were finally analyzed. The prevalence and clinical predictors of HUTT-induced CI syncope were assessed. This study was conducted according to the Declaration of Helsinki and approved by the institutional ethics committees (ID-168/02032021); written informed consent for data collection was obtained from the patients.

### Statistical analysis

Continuous variables were reported as average ± standard deviation, and binary variables as counts and percentages. Between-group comparisons of continuous variables were performed with the Mann–Whitney *U* test after evidence of non-normal distributions obtained with the Shapiro–Wilk test; Pearson’s chi-squared test or Fisher’s exact test (when cells of a contingency table contained frequencies ≤ 5) were used to compare frequencies. Univariate and multivariate analyses were performed to test the association of an asystolic response with HUTT with a set of clinical covariates. The optimal pool of covariates was identified with an automatic backward stepwise procedure and confirmed by forward and mixed forward/backward procedures. *P* to remove variables was 0.2; *p* to include was 0.1. The results have been presented as odds ratios (OR) with 95% confidence interval (ConInt) for each covariate in the model. A value of *P* < 0.05 was considered significant for all measurements. All statistical analyses were performed using STATA software, v.12.0 (Statacorp, TX, USA).

## Results

**Fig. 1:**
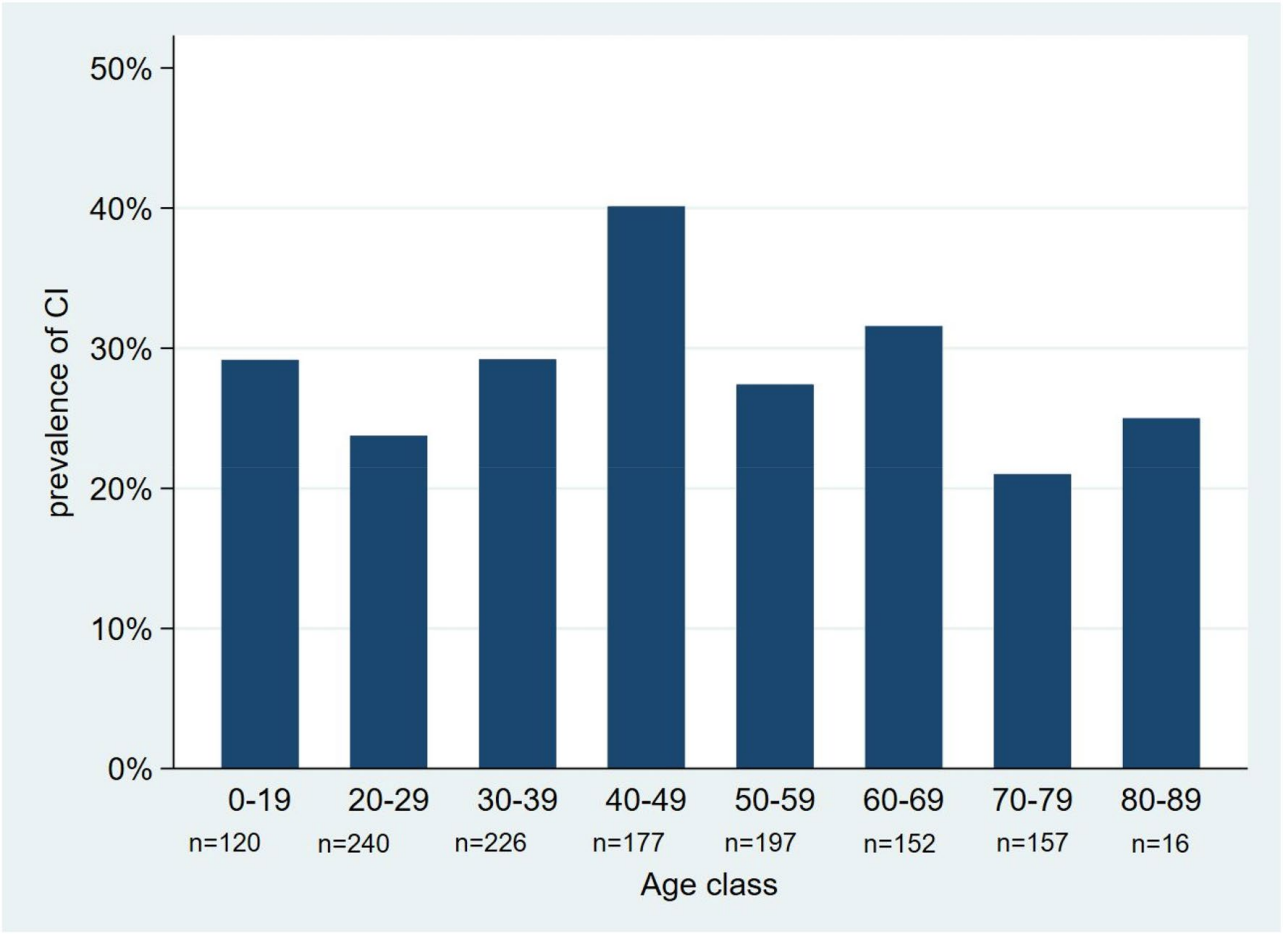
Prevalence of cardioinhibitory (CI) syncope with asystole across different age classes divided by decades

A total of 1285 patients (45 ± 19.1 years; 49.6% male) were enrolled in the present study; among them, 853 patients (66.4%) showed HUTT positivity for reflex neurally-mediated syncope. In particular, 406 (31.6%) patients showed a mixed response, 79 (6.1%) a VD response, and 368 (28.6%) a CI response with asystole to HUTT. Figure 1 shows the prevalence of CI syncope with asystole across different age classes divided by decades. Among the CI syncope group, 68 patients (18.5%) showed the asystole during the passive phase of HUTT. No significant difference in CI prevalence was shown between patients with HUTT positivity during passive versus NTG-potentiated phase (40.5% vs. 43.6%; *P* = 0.47). Male gender, smoking habit, history of hypertension, trauma secondary to syncope, and use of diuretics were more frequent in the cardioinhibitory syncope group, whereas history of situational syncope was more frequent in patients with a non-CI response (Table 1).

**Table 1:** Baseline characteristics of study population

	Overall population (*n*, 1285)	Cardioinhibitory syncope group (*n*, 368)	Non-cardioinhibitory syncope group (*n*, 917)	*P* value^1^
Age (years), mean ± SD	44.4 ± 19.1	43.9 ± 17.9	44.5 ± 19.5	0.77
Male gender, *n* (%)	637 (49.6%)	205 (55.7%)	432 (47.1%)	0.005
Smoking, *n* (%)	202 (15.7%)	88 (23.9%)	114 (12.4%)	< 0.001
Supine Heart Rate (bpm), mean ± SD	72 ± 14	71 ± 14	72 ± 14	0.32
Supine SBP values (mmHg), mean ± SD	122 ± 20	121 ± 20	122 ± 19	0.15
Supine DBP values (mmHg), mean ± SD	75 ± 11	74 ± 11	75 ± 11	0.15
Orthostatic^b^ Heart Rate (bpm), mean ± SD	79 ± 16	79 ± 16	80 ± 16	0.31
Orthostatic^b^ SBP values (mmHg), mean ± SD	121 ± 19	120 ± 19	122 ± 19	0.08
Orthostatic^b^ DBP values (mmHg), mean ± SD	78 ± 10	75 ± 10	76 ± 10	0.1
Hypertension, *n* (%)	188 (14.6%)	72 (19.6%)	116 (12.6%)	0.002
Diabetes mellitus, *n* (%)	50 (3.9%)	17 (4.6%)	33 (3.6%)	0.39
CAD, *n* (%)	26 (2.0%)	12 (3.3%)	14 (1.5%)	0.08
CKD, *n* (%)	24 (1.9%)	6 (1.6%)	18 (2.0%)	0.69
Syncope before HUTT, n, mean ± SD	2.4 ± 0.7	2.4 ± 0.8	2.4 ± 0.79	0.13
Traumatic syncope, *n* (%)	96 (7.5%)	49 (13.3%)	47 (5.1%)	< 0.001
Situational syncope, *n* (%)	124 (9.6%)	22 (5.6%)	102 (11.1%)	0.005
Typical reflex syncope (with prodromes), n (%)	602 (46.8%)	163 (44.3%)	439 (47.9%)	0.24
ACE-I /ARBs, *n* (%)	39 (3.0%)	12 (3.3%)	27 (2.9%)	0.72
Beta-blockers, *n* (%)	22 (1.7%)	9 (2.4%)	13 (1.4%)	0.23
Calcium channel antagonists, *n* (%)	11 (0.9%)	6 (1.6%)	5 (0.5%)	0.09
Diuretics, *n* (%)	39 (3.0%)	28 (7.6%)	11 (1.2%)	< 0.001
Alfa-blockers, *n* (%)	52 (4.0%)	15 (4.1%)	37 (4.0%)	0.97
Insulin, *n* (%)	38(3.0%)	15 (4.1%)	23 (2.5%)	0.15
Oral hypoglicemics, *n* (%)	23 (1.7%)	4 (1.1%)	19 (2.1%)	0.35

*SBP* systolic blood pressure, *DBP* diastolic blood pressure, *CAD* coronary artery disease, *ACE-Is* angiotensin converting enzyme inhibitors, *ARBs* Angiotensin II receptor blockers, *CKD* chronic kidney disease

^a^For continuous variables *P* values are the results of the Mann Witney test to compare cardioinhibitory versus non-cardioinhibitory syncope groups, as all continuous variable resulted non-normally distributed at the Shapiro–Wilks test; for binary variables, the Pearson’s chi-squared test was used, except for calcium channel antagonists and oral hypoglicemics showing frequencies ≤ 5 for which we used the Fisher’s exact test.

^b^Measured during HUTT 2 min after tilting-up

**Fig. 2:**
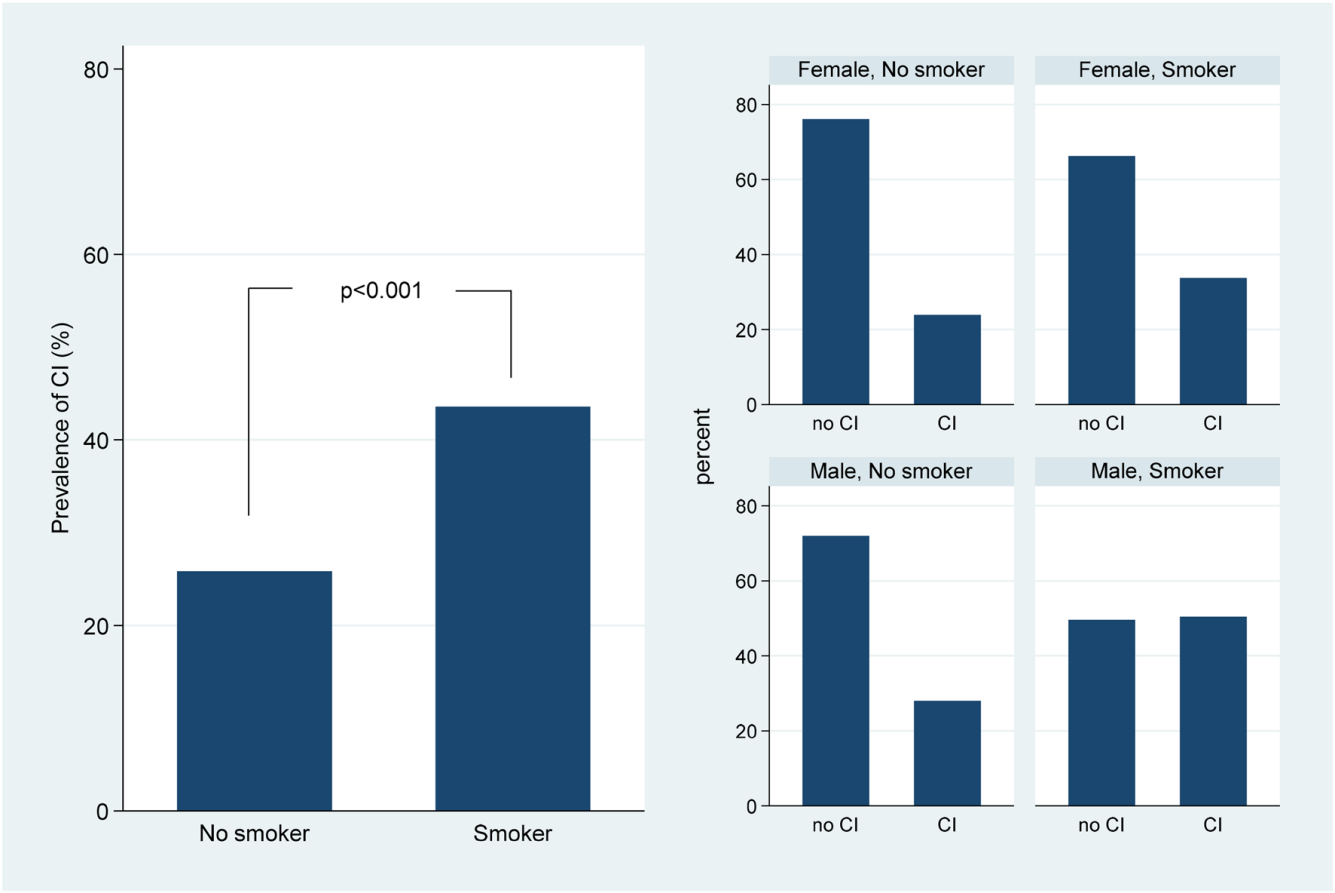
Prevalence of cardioinhibitory (CI) syncope with asystole at head-up tilt test by gender and smoking

At multivariate analysis, male sex, smoking, traumatic syncope and the use of diuretics were independently associated to HUTT-induced cardioinhibitory syncope. Situational syncope inversely correlated with HUTT-elicited asystole (Table 2). Irrespective of gender subgroups, smoking was associated with higher prevalence of CI syncope (*P* < 0.001) (Fig. 2).

**Table 2:** Logistic regression for cardioinhibitory response to HUTT

	Univariate analysis		Multivariate analysis	
	OR (95% ConInt)	*p*	OR (95% ConInt)	*p*
Age (decades)	0.98 (0.92–1.05)	0.60	/	/
Male gender	1.41 (1.11–1.80)	0.005	1.48 (1.14–1.92)	0.003
Smoking	2.21 (1.62–3.01)	< 0.001	2.22 (1.56–3.15)	< 0.001
Heart rate	0.99 (0.98–1.01)	0.43	/	/
Systolic BP values	0.99 (0.99–1.00)	0.40	/	/
Diastolic BP values	0.99 (0.97–1.00)	0.15	/	/
Hypertension	1.68 (1.22–2.32)	0.002	1.38 (0.87–2.01)	0.08
Diabetes mellitus	1.30 (0.71–2.36)	0.40	/	/
CAD	2.18 (0.99–4.75)	0.06	/	/
CKD	0.83 (0.32–2.10)	1.00	/	/
Syncope before HUTT	1.14 (0.97–1.34)	0.12	/	/
Traumatic syncope	2.84 (1.87–4.33)	< 0.001	2.81 (1.79–4.42)	< 0.001
Situational syncope	0.51 (0.31–0.82)	0.005	0.45 (0.27–0.73)	0.002
Typical reflex syncope	0.87 (0.68–1.10)	0.24	/	/
ACE-Is/ARBs	1.11 (0.56–2.22)	0.76	/	/
Beta-Blockers	1.74 (0.74–4.11)	0.20	/	/
Calcium channel antagonists	3.02 (0.92–9.97)	0.07	/	/
Diuretics	6.78 (3.34–13.78)	< 0.001	9.94 (3.83–25.76)	< 0.001
Alfa-blockers	1.01 (0.55–1.86)	0.97	/	*/*
Insulin	1.65 (0.85–3.20)	0.14	/	*/*
Oral hypoglicemic	0.92 (0.86–0.99)	0.03	0.28 (0.07–1.12)	0.07

*ConInt* Confidence interval, *BP* blood pressure, *CAD* coronary artery disease, *ACE-Is* angiotensin converting enzyme inhibitors, *ARBs* Angiotensin II receptor blockers, *CKD* chronic kidney disease

Based on the current recommendations [8], 191 patients (14.9%) with HUTT-induced cardioinhibitory response had indications for dual-chamber cardiac pacing.

## Discussion

The main findings of the present study can be summarized as follows: 28.6% of study population showed a cardioinhibitory response with asystole to NTG-potentiated HUTT. Male sex, smoking habit, history of traumatic syncope, and use of diuretics were independent predictors of HUTT-induced cardioinhibitory syncope. Conversely, situational syncope reduced the probability of cardioinhibitory response to HUTT.

The prevalence of HUTT-induced cardioinhibitory syncope with asystole in our study population is higher than those reported by previous studies, ranging from 4.3% to 19% [3–6]. This wide range may be explained by several factors related to both HUTT methodology, including the drugs used for pharmacological challenge, the time to tilt down, and the clinical features of enrolled patients. In particular, nitroglycerin protocol and younger age are factors favoring HUTT-induced CI response across different studies. CI responses are more rarely observed with passive tilt protocols and with isoproterenol protocols than with nitroglycerin protocols [2, 5]. Our results showed a lower absolute number of CI syncope during the passive phase of HUTT; however, among patients with HUTT positivity, no significant difference in CI prevalence during passive versus NTG-potentiate phase has been shown.

Among 1322 patients with syncope of unknown etiology, Baròn-Esquivias et al. [3] identified 4.3% CI responses at HUTT performed according to the Westminster protocol and 6.5% CI responses at isoproterenol-potentiated HUTT. Recently, Rivasi et al. [5], in a retrospective multicenter study including 5236 patients (mean age 60 ± 22; male 45%) investigated for suspected vasovagal syncope by the Italian HUTT protocol, showed an overall 10% of CI response. Asystolic form was present in 18% of patients < 50 years, and then progressively decreased up to 3% in patients older than 80 years.

The time to tilt down is another main factor influencing the prevalence of CI responses, and it may explain the higher prevalence observed in our study. Contrary to many previous studies [5], which considered the occurrence of pre-syncope a criterion for HUTT interruption, underestimating the total amount of asystole is a further important factor. In the present study, HUTT was continued until complete LOC occurred, indicated by lack of response to vocal stimuli, loss of muscle tonus, and jerking movements. We emphasize the need to consider actual syncope (as defined above) and not just pre-syncope, as the correct diagnostic criteria for HUTT interruption in order to identify the hemodynamic pattern of HUTT response. This methodological approach is of pivotal importance, since the BIOSync trial [10], along with additional evidence from smaller randomized [11] or long-term observational studies [12, 13], provided the basis for the class I indication for the dual-chamber pacemaker in ≥ 40-year-old patients with recurrent, unpredictable syncope recurrence and asystole response to HUTT. In our clinical practice, 14.9% of the study cohort had indications for cardiac pacing according to the current guidelines [8]. Based on these findings, even if HUTT should not be necessary in patients with clinical history of typical reflex syncope, it may provide important pathophysiological evidence of the underlying mechanisms, and can guide appropriate therapy. A subgroup analysis of the ISSUE-3 Trial [14] showed that the asystolic cardioinhibitory response at NTG-potentiated HUTT predicted a similar asystolic form during implantable loop recorder monitoring, with a positive predictive value of 86%.

Previous studies [15], adopting different protocols and including smaller cohorts, showed no significative gender effect on HUTT response; however, it was observed that VD type was slightly higher among females and CI response was numerically higher among males. We have shown that males are more likely to be at increased risk of HUTT-induced CI syncope compared to females.

Contrary to previous studies [16], we did not show any association between CI syncope and age. The timing of tilt down may have played a role in masking the reduced CI prevalence, since a recent meta-analysis of 55 studies, including > 4.300 patients undergoing HUTT, revealed that dependence of CI prevalence on age is remarkably attenuated if the table was tilted down at the time of complete loss of consciousness [17], as we did in our study.

Very few data are available about the relationship between smoking and HUTT response. It has been proposed that endothelial function and inappropriate peripheral vasomotion may have a significant role in the pathogenesis of neurally-mediated syncope [18], and may predict a negative response to HUTT [19]. Moreover, low plasma levels of adrenomedullin and endothelin-1 predict cardioinhibitory response during vasovagal reflex in adults over 40 years of age [20]. In our experience, smoking seems to be a marker of greater susceptibility to orthostatic stress leading to cardioinhibitory HUTT response. This effect was observed across levels of all other predictors identified in the multivariate analysis, and smoking was associated with higher prevalence of CI syncope, irrespective of gender subgroups. The association of VD reflex syncope with chronic vasoactive drug therapy is a frequent clinical problem, especially in the elderly. The use of thiazides has been shown to be an independent predictor for syncope even after correction for age and the presence of hyponatremia and hypokalemia [21].

The recurrence of syncope can be reduced by discontinuing/reducing vasoactive therapy in most elderly patients affected by syncope [22]. No previous studies have evaluated the association between pharmacological therapy and the type of HUTT response. Our data suggest a positive association between the use of diuretics and HUTT-induced cardioinhibitory response. It has been shown that the incidence of trauma did not differ among patients with a syncope or a non-syncopal transient LOC [23]. However, among patients with suspected reflex syncope, our data suggest that the history of traumatic syncope may predict the HUTT-induced CI response. The short duration of prodrome and the rapid onset of asystole might explain this association. This finding has practical implications, since the trauma should be advocated as a red flag for a severe form of reflex syncope in need for fast-track access to HUTT, while the early detection of underlying mechanisms may lead to the application of a specific treatment in order to prevent both recurrences and associated further physical injuries. Conversely, the history of situational syncope seems to reduce the probability of HUTT-induced cardioinhibitory syncope, enhancing the previous evidence of the relatively marginal role of CI in the mechanism of situational syncope [24].

### Limitations

Our results should be interpreted considering the limitations related to the study’s retrospective, observational, single-center nature. The high percentage of CI response among our study population was not driven by the HUTT positivity rate; indeed, we confirm the overall positivity rate of the nitroglycerin protocol showed by Forleo et al. [2] in a recent systematic literature review including 4361 patients with syncope. The temporal relationship of asystole to LOC onset was not evaluated through video-recording. Therefore, we cannot exclude the inclusion of patients with HUTT-induced asystole occurred too late to have been the primary cause of the LOC. However, the expertise of our syncope nurse specialist and the use of the tilt table with short tilt down time should have reduced this probability. The lack of association between CI syncope and age might be related to the concomitant effect of other covariates. In particular, since patients with a smoking habit, traumatic syncope, and diuretics were significantly older than others, and these factors were also the strongest predictors of CI, the contrary effect of age might be counterbalanced.

## Conclusions

The cardioinhibitory syncope with asystole induced by NTG-potentiated HUTT is more frequent than previously reported. Male sex, smoking habit, history of traumatic syncope, and use of diuretics were independent predictors of HUTT-induced cardioinhibitory response. Conversely situational syncope seems to reduce this probability. These data might be useful to identify patients in need of fast-track access to HUTT for the early detection of cardioinhibitory syncope with asystole.

## Data Availability

The data that support the findings of this study are available from the corresponding author, upon reasonable request.
